# The First Case of Huntington’s Disease like 2 in Mali, West Africa

**DOI:** 10.5334/tohm.859

**Published:** 2024-04-02

**Authors:** Abdoulaye Bocoum, Madani Ouologuem, Lassana Cissé, Fahmida Essop, Souleymane dit Papa Coulibaly, Nadine Botha, Cheick A. K. Cissé, Alassane dit Baneye Maiga, Amanda Krause, Guida Landouré, The H3Africa consortium

**Affiliations:** 1 Facultéde Médecine et d’Odontostomatologie, USTTB, Bamako, Mali; 2 Service de Médecine, Hôpital du Mali, Bamako, Mali; 3 Service de Neurologie, CHU Point “G”, Bamako, Mali; 4 Division of Human Genetics, National Health Laboratory Service and School of Pathology, Faculty of Health Sciences, The University of the Witwatersrand, Johannesburg, South Africa; 5 Service de Psychiatrie, CHU Point “G”, Bamako, Mali

**Keywords:** HDL2, JPH3, Mali, West Africa

## Abstract

**Background:**

Huntington’s disease like 2 (HDL2) has been reported exclusively in patients with African ancestry, mostly originating from South Africa.

**Case report:**

We report three patients in Mali including a proband and his two children who have been examined by neurologists and psychiatrists after giving consent. They were aged between 28 and 56 years old. Psychiatric symptoms were predominant in the two younger patients while the father presented mainly with motor symptoms. Genetic testing identified a heterozygous 40 CTG repeat expansion in the Junctophilin-3 (JPH3) gene in all three patients.

**Discussion:**

This study supports the hypothesis that HDL2 may be widely spread across Africa.

**Highlights:**

We report here the first case of HDL2 in West Africa, suggesting that HDL2 is widely spread across African continent, and increasing access to genetic testing could uncover other cases.

## Introduction

Huntington’s disease-like 2 (HDL2) is an autosomal dominant neurodegenerative disorder characterized by its clinical similarity with Huntington’s disease (HD) and caused by a CTG repeat expansion in the exon 2 of the junctophilin-3 (*JPH3*) gene on chromosome 16q24.3 [1,2].

Since its first description in 2001, seventy cases had been reported up to 2017 around the world, mostly in South Africa and the United States. with all the patients having established or suspected African ancestry [3,4].

We report here three confirmed cases of HDL2 in a family from West Africa, supporting a more widespread distribution of the disease in Africa.

## Case report

Patients were seen under a research protocol approved by the ethical committee of the Faculté de Médecine et d’Odonto-stomatologie (FMOS), Mali. The clinical examination was performed by a multidisciplinary team including neurologists and psychiatrists. Brain MRI and the limited blood chemistries available in Mali were performed to exclude other causes of the phenotype.

DNA was extracted from peripheral blood for genetic testing that was performed in the Division of Human Genetics, National Health Laboratory Service (NHLS) & The University of the Witwatersrand, South Africa. PCR amplification and size differentiation on the Genetic analyzer was performed to test for the *HTT* and *JPH3* expansions. Repeat sizes were classified according to GenBank Accession Number NM_002111.8 and NM_020655.4 for HTT and JPH3 respectively. Repeat sizes are described and interpreted in line with EMQN best practice guidelines [5].

Three affected patients (two males and one female) from one family were tested. The family originated from the Timbuktu region (northern region of Mali) and identify with the Songhai ethnic group. The patients were aged between 26 and 52 at the onset of their symptoms and between 28 and 56 years at the time of diagnosis. The proband (III-5) is a 56-year-old male smoker with a four-year history of uncontrolled movements in his face, limbs and trunk, causing instability when he is seated and walking. At the same time, his relatives have noticed behavioral changes, including irritability. His symptoms have worsened progressively, leading to a severe disability.

**Figure 1:**
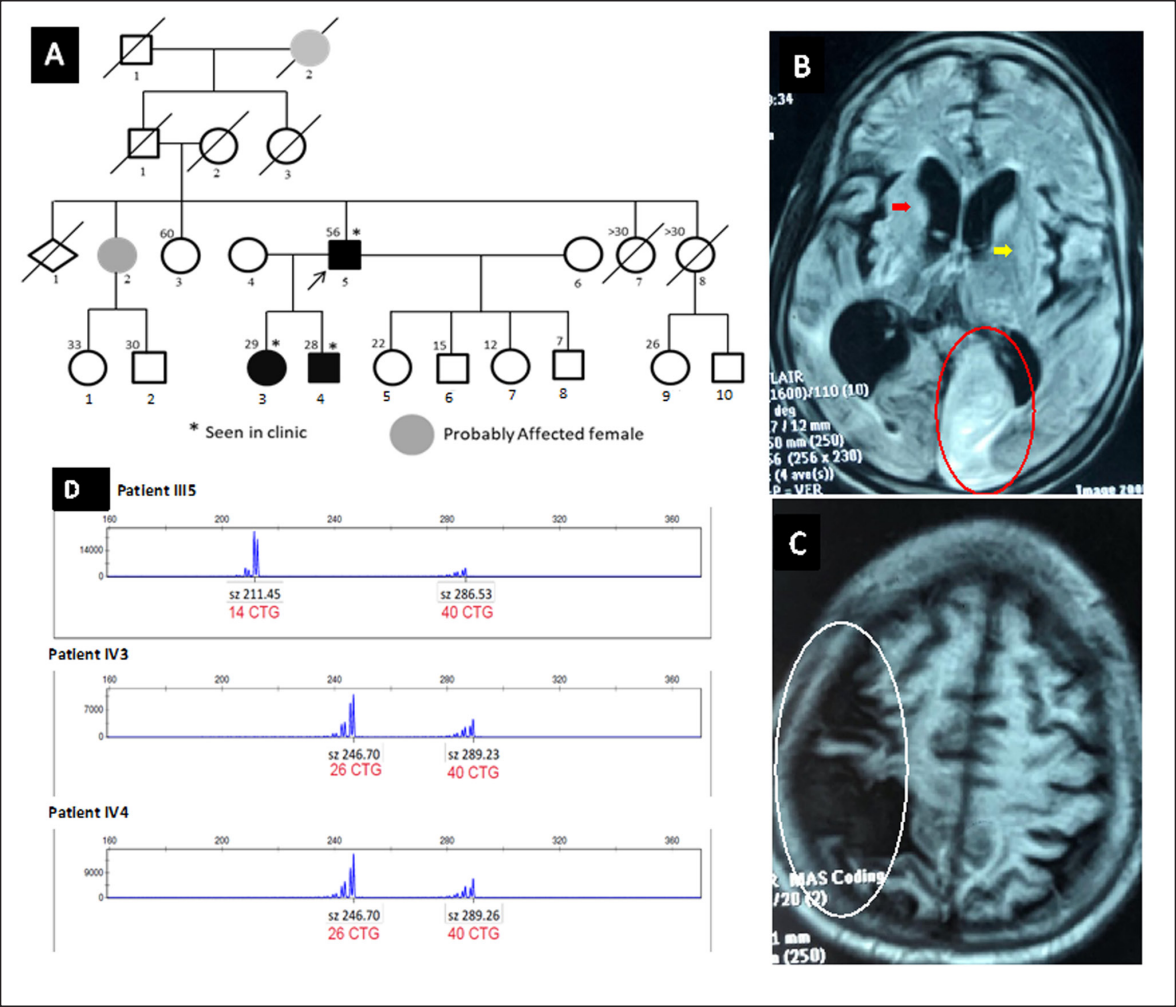
Phenotypic features of family with HDL2: **A)** Pedigree showing the dominant pattern of inheritance and brain MRI of the proband **B)** T2 FLAIR sequence showing an hyperintensity in the territory of the left posterior artery cerebral (red circle) and bilateral atrophy of caudate and lenticular nuclei (red and yellow arrows), and **C)** T1 FLAIR sequence showing a hypo-intensity in superficial territory of the right sylvian artery (white circle). **D)** Genetic results showing the abnormal CTG repeat expansion (40) in the *JPH3* gene.

Several other family members are reported to have presented with similar symptoms, including his paternal grandmother who was “shaking” (individual I-2), an older sister (III-2) who has a mood disorder and two of his children, including a daughter and a son (IV-3 and IV-4) both predominantly afflicted by psychiatric symptoms (Figure 1).

Neurological examination of the proband (III-5) found irregular and altered speech and severe generalized chorea with oro-facial, trunk and limbs involvement. Oculomotor movements were slow in their initiation and velocity. He had an unsteady gait with a tendency to fall, and he was unable to tandem walk. There was a rigidity of his upper and lower limbs as well as a generalized bradykinesia. No muscle weakness and sensory impairment was noted, but he had brisk reflexes in all four limbs. The Total Motor Score (TMS) of the United Huntington’s Disease Rating Scale (UHDRS) was 74 and the Mini Mental State Examination (MMSE) score was 27.

Brain MRI showed bilateral signal anomalies consisting of hypo-intensity in the superficial territory of the right middle cerebral artery in T1 FLAIR and an hyperintensity in the territory of the left posterior cerebral artery in T2 FLAIR sequences; suggesting multiple ischemic strokes. Additionally, cortical and basal ganglia atrophy was seen (Figure 1B and C).

The patient had initially been treated with Olanzapine up to 20 mg per day with no improvement. Then Tetrabenazine (12.5 mg twice per day initially, then increased to 25 mg twice a day) was introduced, improving his symptoms considerably during the first three months. However, symptoms worsened progressively later, leading to severe generalized chorea with inability to speak and to walk alone as well as irritability (Video 1).

**Video 1:** A 56-year-old patient with severe generalized chorea and inability to speak.

His 29-year-old daughter (IV-3) was seen for a year-long progressive speech impairment and mood disorder. She has been under psychiatric management for severe depression that was diagnosed four years previously with multiple suicide attempts of . Her brother has reported that she has difficulty with social integration and alternates periods of apathy and irritability. Clinical examination found a well-oriented patient with a slight speech impairment consisting of hesitation and breaks during the speech as well as the need to repeat some words, normal oculomotor functions, diffuse brisk osteotendinous reflex, and no other motor and sensory impairment. She had a depressed mood and cried during the examination. She was treated with Amitriptyline (50 mg daily) and Bromazepam 1 mg a day with no change in the course of the disease.

His 28-year-old son (IV-4) was seen for an unsteady gait and difficulties with speech. These symptoms had appeared some months ago and were worsening progressively. He had been recently admitted to the psychiatry department for alcohol and tobacco addiction and is undergoing withdrawal process under medical supervision. The clinical examination found slight and temporary abnormal movements in the peri-buccal region and a rapid tremor in his hands, of which he was unaware. None of his features are consistent with his alcohol intake or withdrawal. No cognitive impairment was found.

Genetic testing of the *Huntington (HTT*) gene in the proband found a normal CAG repeat size of both alleles (15 and 19 CAG repeats respectively). Testing for HDL2 was then performed and found a heterozygous abnormal expansion of CTG repeats at the *JPH3* locus (40 CTG repeats expansion on the mutated allele and 14 on the normal allele), confirming the diagnosis of HDL2. Subsequently, testing of the two children has confirmed the HDL2 diagnosis with 40 CTG repeats expansion on the mutated allele and 26 on the normal allele in both.

The clinical and genetic findings are summarized in Table I.

**Table I:** Clinical and genetic features of patients with HDL2 expansion.


PATIENT	GENDER	AGE (YEARS)	AGE AT ONSET (YEARS)	FIRST SYMPTOMS	MAIN CLINICAL FEATURES	CTG REPEATS (N/M)

III-5	M	56	52	Involuntary movements	Generalized chorea, speech and gait impairment, irritability	14/40

IV-3	F	29	26	Speech impairment and mood disorder	Speech impairment, mood disorder	26/40

IV-4	M	28	28	Unsteady gait	Peri-buccal chorea, rapid hand tremor	26/40


N/M: normal/mutated; CTG: Cytosine-Thymine-Guanine.

## Discussion

HDL2 is a neurodegenerative disease characterized by a movement disorder, progressive dementia, parkinsonism, and psychiatric symptoms. It is the most common HD phenocopy in South Africa, South America, the USA, and some part of Europe, and is due to an expanded CTG repeats in the *JPH3* gene [1,6,7,8,9]. HD and HDL2 have similarities in terms of clinical characteristics including symptoms, age at onset, progression, and outcome, though an earlier onset with more prominent dysarthria and dystonia has been reported in HDL2 patients [2].

On the African continent, with the exception of one case reported from southern Morocco from a region populated with people of Black African ancestry, all cases have been reported in southern Africa including South Africa and Botswana [4]. In addition, cases have been reported in Europe, North America and South America, but all are suspected to have African ancestry [10,11].

To our knowledge, this is the first family with HDL2 reported on the African continent outside southern Africa. The family described here is from Timbuktu where 33.3% of our previously reported HD cases originated [12]. Moreover, the family has a Songhai ethnic background, a Bantu-speaking ethnic group that probably originated from the hypothetic Bantu expansion across sub-Saharan Africa as did many of the people in South Africa, suggesting a possible common founder [13]. The same studies suggest that many African Americans also originate from the Bantu speaking community, consolidating the common founder effect theory for HDL2 variants. Further haplotype studies including patients from these regions could elucidate this assertion, as all patients studied to date with HDL2 have a common founder haplotype [14].

Although recall of first symptoms is sometimes difficult for patients with chronic diseases, the symptoms in the proband appear to have started in his early fifties while they have started earlier in his offspring (late twenties) despite all carrying the same abnormal repeats expansion size. This suggests on one hand that there is a relative stability of that gene region during the transmission from one generation to the next, but on the other hand that there could be other disease modifying factors involved. However, it could be that behavioral changes in the proband may have gone unnoticed for several years. Abnormal movements and psychiatric symptoms are also pathognomonic in the classical presentation of HD, but the age at onset is earlier in HDL2, on average compared to HD patients seen in the same population (mean age at onset 49.7 years) [11]. Therefore, it is difficult to make any distinction based on clinical findings when comparing HD to HDL2, the genetic testing being key to making the diagnosis [2].

A vascular chorea has been considered for the proband based on his risk factors and evidence of stroke, but the lesions present are in regions that do not match the topography of the patient’s symptoms. The patient may have had unrelated silent strokes.

For the two children, despite the social difficulties they have had during their childhood, their symptoms likely reflect HDL2 and no other pathology. The neuropsychiatry symptoms may be prominent early in HDL2 [15].

Our report suggests that HDL2 should be tested in all patients with African ancestry presenting with an HD phenotype and with no other apparent causes of their symptoms. Furthermore, it supports the suggestion that HDL2 may be present in other parts of Africa.
